# When Sutures Fail: Extrusion of an Inspire Device and a Hip Prosthesis in the Same Patient

**DOI:** 10.7759/cureus.89280

**Published:** 2025-08-03

**Authors:** Kalin R Sorenson, Cameron Robins, Keala Verigan, Nicholas Gerber, Louis Musso

**Affiliations:** 1 Otolaryngology, Rocky Vista University College of Osteopathic Medicine, Ivins, USA; 2 Surgery, Rocky Vista University College of Osteopathic Medicine, Ivins, USA

**Keywords:** ethibond, hip prosthesis, implant extrusion, inspire device, suture hypersensitivity

## Abstract

Implant extrusion is a rare but serious complication typically linked to infection, poor wound healing, or material intolerance. This case report describes a 79-year-old female patient who experienced extrusion of both a hip prosthesis and an Inspire hypoglossal nerve stimulator (Inspire Medical Systems, Inc., Minnesota, USA), each event associated with the use of Ethibond (Ethicon, Johnson & Johnson, Georgia, USA) sutures. The patient had a history of obstructive sleep apnea refractory to continuous positive airway pressure (CPAP) therapy and underwent Inspire device implantation. Postoperatively, she developed a sterile fluid collection and wound dehiscence, leading to visible extrusion of the device. Notably, she had previously experienced a near extrusion of a hip prosthesis, also linked to Ethibond. In both cases, the infectious workup was negative. Following explantation and reimplantation of the Inspire device using Monocryl and Prolene sutures (both by Ethicon, Johnson & Johnson, Georgia, USA) and the application of a dermal graft, the patient achieved successful healing without recurrence. This case underscores the importance of considering suture-related foreign body reactions in patients with recurrent implant extrusion and no evidence of infection. Surgeons should remain vigilant in selecting suture materials, especially in patients with a history of adverse reactions, and future research should explore suture-tissue interactions to guide safer implant practices.

## Introduction

Implant extrusion is a rare but significant complication that can compromise the success of device-based therapies. A variety of factors contribute to this adverse outcome, including mechanical stress from trauma or repetitive manipulation, infection, poor surgical technique, and inadequate tissue vascularization or quality. These factors can compromise tissue integration and support, leading to exposure or complete loss of the implant. While infection and poor wound healing are common contributors, less frequently, suture-related reactions may lead to implant failure. 

While the integrity and long-term stability of medical implants are critical for favorable patient outcomes, an underexplored variable in implant success is the choice of the suture material. Although the current literature on implant extrusion predominantly focuses on infections, mechanical failure, and vascular compromise, the role of suture-induced tissue reactions has received limited attention. Reports of implant extrusion are most frequently associated with ophthalmic, breast, dental, and cochlear implants [1-3], with only minimal discussion of how different suture types might influence these events.

Ethibond (Ethicon, Johnson & Johnson, Georgia, USA), a nonabsorbable, braided polyester suture coated with polybutylate, is widely used in surgical practice due to its high tensile strength and minimal knot slippage [4]. However, when compared to other braided sutures, Ethibond may exhibit lower abrasion resistance and reduced tensile strength under certain conditions [4,5].

Beyond its mechanical properties, Ethibond - like many braided sutures - has been implicated in inciting a pro-inflammatory tissue response. This immune activation can promote osteoclast differentiation and bone resorption, potentially contributing to implant instability or extrusion, especially when compounded by bacterial colonization [6].

Histologic evidence from a 2019 sheep shoulder model demonstrated tissue separation and foreign body reaction associated with Ethibond sutures, suggesting that certain host-suture interactions may increase the risk of implant extrusion [7]. Despite such findings, the literature remains sparse on studies directly evaluating suture material as a primary contributor to extrusion risk.

This case report describes a 79-year-old former smoker with a history of obstructive sleep apnea (OSA) who underwent placement of an Inspire hypoglossal nerve stimulator (Inspire Medical Systems, Inc., Minnesota, USA). Despite the absence of infection, she developed a fluid collection and wound dehiscence, ultimately resulting in implant extrusion. Notably, the patient also had a prior history of near extrusion of a hip prosthesis, similarly associated with the use of Ethibond suture. The patient's history as a former smoker may have further contributed to impaired wound healing, but the recurrence of extrusion with linkage to Ethibond suture suggests an underlying material-related reaction. 

This case underscores the importance of recognizing suture intolerance as a potential mechanism of implant failure and highlights the need for further investigation into suture-induced inflammatory responses and their clinical implications.

## Case presentation

A 79-year-old former smoker with a history of severe OSA presented with persistent intolerance to continuous positive airway pressure (CPAP) therapy despite multiple attempts at desensitization and mask adjustments. Her apnea-hypopnea index (AHI) was 50.8 events per hour, and she experienced unrefreshing sleep, daytime fatigue, and witnessed apneas. A drug-induced sleep endoscopy (DISE) demonstrated anteroposterior velum and tongue base collapse without complete concentric collapse, making her a candidate for hypoglossal nerve stimulator (HGNS) implantation. After discussing therapeutic options, she elected to undergo placement with the Inspire device.

In March 2022, the patient underwent successful implant surgery for the Inspire device with placement of a right hypoglossal nerve lead and a right-sided chest wall pulse generator. During follow-up, she reported satisfactory device function with subjective improvement in sleep quality and reduction in daytime fatigue. However, she developed progressive erythema, swelling, and fluctuance over the incision site on the chest. Despite conservative management, the incision dehisced laterally, resulting in the exposure of the implant. Notably, multiple cultures were negative for bacterial growth and no evidence of infection was found. Clinical documentation of the extrusion event was limited, and no photograph was obtained. However, multiple progress notes consistently described a localized dehiscence with a visible implant and fluid drainage. The area of dehiscence remained stable in size but failed to re-epithelialize over time. Despite the implant being noted as 'secure and in place,' extrusion progressed and ultimately necessitated removal.

A thorough review of her history revealed a prior near extrusion of a hip prosthesis, also associated with the use of Ethibond sutures. Given the absence of infection and the recurrent nature of the extrusion, a suture material reaction was suspected. The Inspire device was explanted in June 2022.

In July 2022, the patient underwent reimplantation of the Inspire device on the left side using Monocryl and Prolene sutures (both from Ethicon, Johnson & Johnson, Georgia, USA), with the addition of a dermal graft for reinforcement. Her postoperative course was uncomplicated, and she resumed Inspire therapy without further issues. Her AHI improved to 3.9 events per hour, and her oxygen desaturation index (ODI) decreased from 48.3 to 14.5, subsequently normalizing to 3.9.

**Figure 1:**
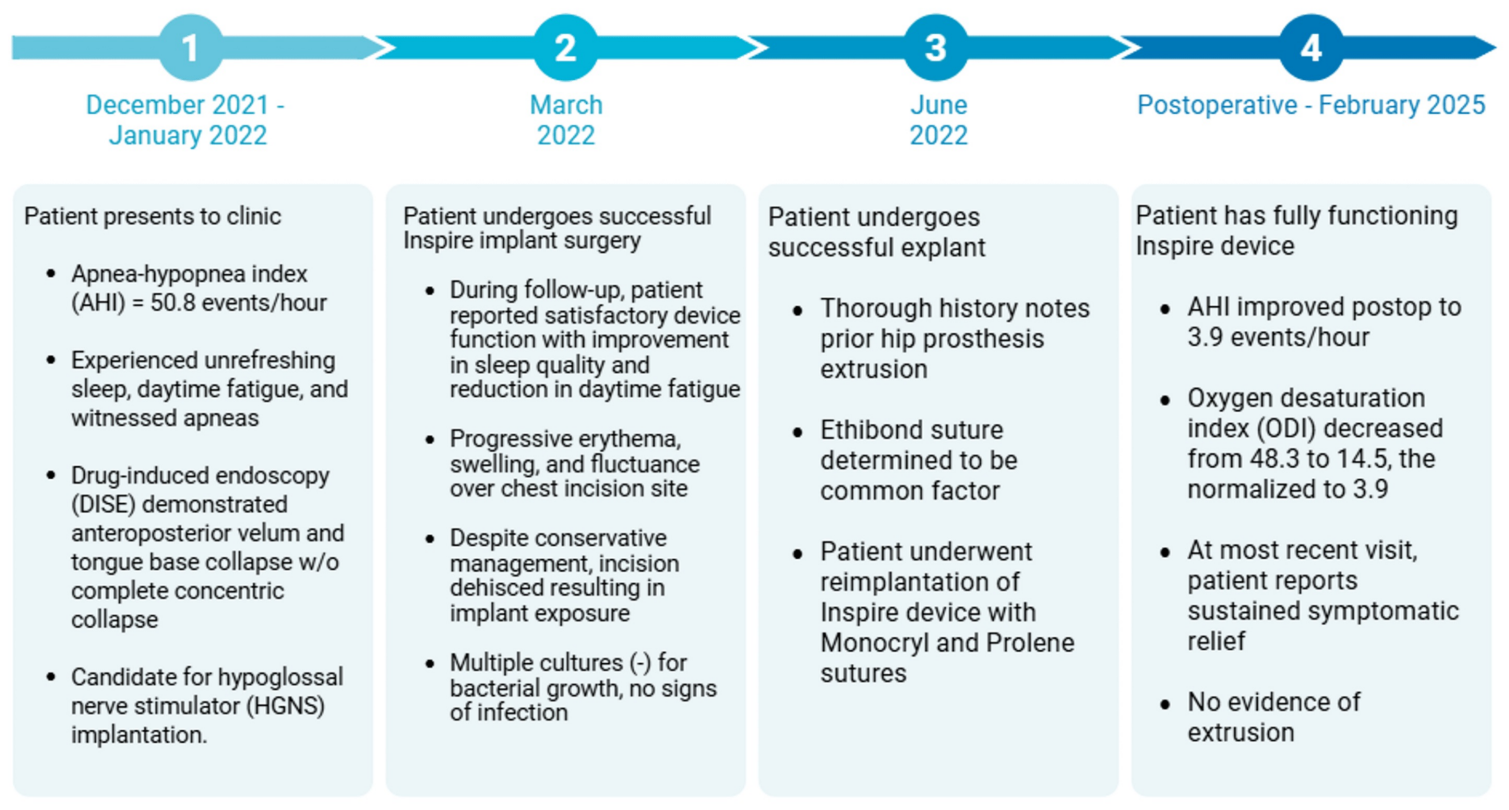
A timeline starting from when the patient first presented to the clinic for her sleep apnea to the most recent postoperative condition

At her most recent follow-up (Figure 1), the patient reported sustained symptomatic relief with no evidence of recurrence.

This case underscores the importance of considering suture material sensitivity in patients with unexplained implant extrusions, particularly when infection has been excluded.

## Discussion

Implant extrusion is a multifactorial complication influenced by both patient-specific and surgical variables. Key contributors include infection, chronic inflammation, mechanical stress at the implant site, tissue ischemia, and foreign body reactions to suture or implant materials. Suture characteristics, such as whether the suture is absorbable or nonabsorbable, monofilament or braided, and synthetic or biologic, may significantly affect tissue compatibility and healing. Additionally, patient-related factors such as smoking, diabetes, immune status, nutritional deficiency, and prior surgical history can further compromise wound integrity. Recognizing how these variables interact is critical to understanding the etiology of extrusion and guiding preventive strategies.

Types of suture

Surgical sutures are categorized as absorbable or nonabsorbable, with origins either synthetic or biological, each suited for specific clinical scenarios. Absorbable sutures degrade naturally, making them ideal for internal tissues, while nonabsorbable sutures provide lasting support for external or load-bearing applications. Table 1 gives a breakdown of commonly used sutures and what they are used for.

**Table 1:** Classification and characteristics of common surgical sutures [8]

Suture type	Suture name	Material type	Common uses
Absorbable sutures	Catgut (Plain/Chromic)	Biological	Superficial closures (e.g., oral mucosa)
	Polyglycolic Acid (Dexon)	Synthetic	Subcutaneous closures in general surgery
	Polyglactin 910 (Vicryl)	Synthetic	Soft tissue approximation in abdominal surgeries
	Polydioxanone (PDS)	Synthetic	Fascial closures needing prolonged support
Nonabsorbable sutures	Polyester (Ethibond)	Synthetic	Cardiovascular or orthopedic repairs
	Nylon (Ethilon)	Synthetic	Skin closures, neurosurgery
	Polypropylene (Prolene)	Synthetic	Hernia repairs, permanent support
	Silk	Biological	Delicate procedures (e.g., ophthalmic surgery)

Sutures also come in three forms: monofilament, braided, and barbed. Monofilament sutures are single-stranded, strong, and have low tissue resistance and infection risk, but are less flexible, harder to handle due to structure memory, and have poorer knot security. They are favored for continuous sutures, skin, tendon, vascular, and microsurgery. Braided sutures have multiple strands woven together, offering greater tensile strength, flexibility, and easier handling, though they cause more tissue drag. They are preferred in high-tension or fragile tissues, such as in cardiac surgery and prosthetic fixation. Barbed sutures are made of monofilaments with surface barbs that anchor tissue without knots, reducing suturing time and reinforcing the suture line; especially useful in laparoscopic and robotic surgeries [8].

Implant and suture extrusion

Implant extrusion is a rare but significant complication following device implantation. While infection is the most common etiology, non-infectious causes such as foreign body reactions or hypersensitivity to suture materials should also be considered. This case highlights the importance of recognizing suture-related complications, particularly in patients with a history of a prior implant extrusion without evidence of infection.

Ethibond, a braided polyester suture, is widely used in surgical procedures due to its tensile strength and durability. Its braided structure can harbor bacteria and provoke exaggerated inflammatory responses, even in the absence of infection [9,10]. In the present case, the patient experienced the extrusion of both a hip prosthesis and an Inspire hypoglossal nerve stimulator, with the Ethibond suture implicated in both instances. Despite sterile cultures and a negative infectious workup, the patient exhibited recurrent wound dehiscence and seroma formation. This clinical pattern strongly suggested a suture-related foreign body reaction.

Pathophysiology

Suture hypersensitivity is an immunologic reaction caused by the body perceiving the suture as a foreign body or antigen. This hypersensitivity leads to overreaction from the body and exaggerated immune responses, which causes inflammation, erythema, and the potential to prevent wound healing and an increased likelihood of wound infection, dehiscence, or extrusion as seen in this case [11].

Suture reactions can present with localized erythema, edema, and fluid collection, often resembling infection. However, the distinguishing features of a suture reaction from an infection include the absence of systemic signs of infection, negative cultures, and recurrent wound complications despite antibiotic therapy. In this case, multiple sterile cultures and the absence of purulence supported the diagnosis of a non-infectious suture reaction. Overall tissue response varies depending on factors such as the suture material used (absorbable vs non-absorbable, synthetic vs biologic), type of tissue receiving the sutures, and the quality of the immune system [12]. Cutaneous responses to suture materials are broadly classified into immediate-type (innate) and delayed-type (adaptive) reactions, mediated by Immunoglobulin E (IgE) and T cells, respectively.

Innate Response

The initial immune response is uniform across suture types and typically manifests within the first five to seven days following implantation, consisting of acute inflammation [12]. This process is thought to represent a combination of both innate defense and an early component of the Type IV hypersensitivity (delayed) reaction. This adaptive reaction is driven by the foreign body nature of sutures, leading to macrophage-mediated cellular responses [12]. With continued exposure, macrophages differentiate into epithelioid histiocytes, promoting the development of granulomatous inflammation.

Granulomas, aggregates of epithelioid histiocytes and lymphocytes, arise through strong T-cell activation and subsequent cytokine production. Absorbable sutures are ultimately degraded via local tissue reactions. However, non-absorbable sutures such as Ethibond persist indefinitely. Over time, the chronic presence of these materials stimulates fibroblast proliferation and encapsulation within fibrous tissue [13]. In rare cases, failure of complete encapsulation results in persistent granulomatous inflammation. Supporting this mechanism, a prior case study involving pacemaker suture extrusion revealed histopathologic findings of chronic granulomatous inflammation [14].

Delayed Response

Delayed-type hypersensitivity requires a two-signal activation model: the first involves antigen-presenting cells (APCs) sensitizing naive T cells, and the second, upon re-exposure, triggers T-cell activation, clonal expansion, and cytokine-mediated tissue injury [11].

**Figure 2:**
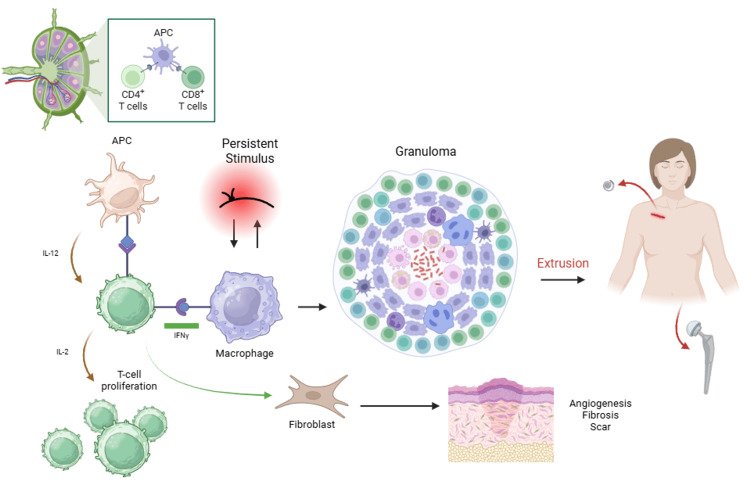
A depiction of the sequence of T-cell activation from an APC that can lead to an extrusion, as in this case, or scarring APC: Antigen-presenting cell. Figure created with BioRender.com. Used with permission under an academic subscription.

In the present case, extrusion of both a hip prosthesis and an Inspire device (Figure 2) occurred following the use of Ethibond sutures, suggesting the patient may have been previously sensitized to the material.

Subsequent surgeries likely reactivated a delayed hypersensitivity response, culminating in suture degradation, disruption of soft tissue fixation, and device extrusion.

Moreover, delayed regional tissue responses must be considered even years after implantation. Lovric et al. (2018) demonstrated in a murine airpouch model that suture wear particles, particularly from FiberWire, elicited a significantly heightened inflammatory response compared to intact sutures [15]. This response included increased multinucleated giant cell formation and elevated expression of matrix metalloproteinases (MMP-1, MMP-2, MMP-9, and MMP-13), key enzymes implicated in extracellular matrix degradation. These findings highlight the potential for suture wear to exacerbate chronic inflammation, ultimately contributing to wound dehiscence and implant extrusion. More research should be done to evaluate if this reaction is common in all braided types of suture, or if this is unique to FiberWire due to its silicone coating while Ethibond consists of polybutylate.

Management

Management of suture-related complications often necessitates removal of the offending material and reimplantation using alternative sutures. In this case, reimplantation of the Inspire device using Monocryl (a synthetic absorbable suture) to anchor the device and Prolene (a monofilament non-absorbable suture) to reapproximate deeper tissue layers and subcutaneous tissue closure, along with dermal graft reinforcement, resulted in successful wound healing without further complications. The patient's subsequent clinical stability and improvement in sleep metrics underscore the effectiveness of this strategy.

Further research is warranted to elucidate the incidence and mechanisms of suture-associated complications, particularly in predisposed individuals. Improved identification of at-risk patients and selection of biocompatible suture materials may enhance surgical outcomes and reduce implant failure rates.

Other considerations

Other contributing factors to implant extrusion must also be considered, including impaired wound healing associated with comorbidities such as diabetes, a history of smoking, poor tissue perfusion, and mechanical stresses imposed by the implanted device itself. These external influences can significantly impact surgical outcomes and should not be overlooked. A thorough preoperative evaluation of patient history and risk factors is critical to minimize the likelihood of extrusion and optimize postoperative healing.

Key learning points

This case underscores several important clinical insights. First, the selection of suture material is critical, as the choice of suture can greatly influence implant stability and long-term surgical outcomes. Braided, non-absorbable sutures, in particular, pose a higher risk due to their ability to harbor bacteria and elicit stronger chronic inflammatory responses when compared to monofilament sutures. These reactions may contribute to delayed complications such as granuloma formation, tissue breakdown, and ultimately implant extrusion. Therefore, understanding and anticipating foreign body responses is essential for preserving implant integrity over time.

Additionally, thorough preoperative evaluation is vital, especially in patients with a history of device extrusion. A careful review of prior surgical outcomes and possible material sensitivities can inform safer, more individualized surgical planning. In complex cases of recurrent extrusion, a multidisciplinary approach involving specialties such as otolaryngology, orthopedics, and plastic surgery is often necessary to optimize management strategies for explantation, revision, and reimplantation. Finally, this case highlights the need for future research into suture performance, particularly in implant-based procedures. Innovations such as anti-inflammatory coatings or bioresorbable materials may offer promising avenues to reduce the incidence of suture-related complications.

## Conclusions

This case illustrates the rare but impactful complication of suture-related implant extrusion. The patient’s history of recurrent extrusion involving both a hip prosthesis and an Inspire device, with negative infectious workup, pointed to a foreign body reaction to Ethibond sutures. Successful reimplantation using Monocryl and Prolene sutures, along with the use of a dermal graft, led to resolution without further complications. During the last office visit in February 2025, the patient reported no complications and good surgical site healing. This case suggests that alternative suture choices or adjunctive measures, such as dermal grafts, may mitigate the risk of recurrent extrusion. Although the precise mechanism by which Monocryl (absorbable) and Prolene (non-absorbable) prevented extrusion while Ethibond did not remains unclear, it may be related to their monofilament structure and the body's differential response to monofilament versus braided sutures. More research should be done to identify the mechanism behind the extrusion of different suture materials and how it relates to the rejection of devices placed within patients. Cases of patients with one or more device rejections should be studied to consider further instances of patient rejection of the suture(s) used rather than rejection of the device that was placed.

This case also emphasizes the importance of thorough preoperative evaluation in patients with a history of implant extrusion. Surgeons should consider prior adverse reactions to surgical materials when planning subsequent procedures. They should also maintain a high index of suspicion for suture-related reactions in cases of unexplained implant extrusion, particularly when infection is not evident. Selecting appropriate suture materials and considering adjunctive measures may help mitigate recurrence in patients with prior adverse suture reactions. Overall, this case further underscores the importance of personalized surgical planning and postoperative vigilance to ensure optimal patient outcomes.
